# Perceptual Soft End-Effectors for Future Unmanned Agriculture

**DOI:** 10.3390/s23187905

**Published:** 2023-09-15

**Authors:** Weikang Ye, Lin Zhao, Xuan Luo, Junxian Guo, Xiangjiang Liu

**Affiliations:** 1 College of Biosystems Engineering and Food Science, Zhejiang University, Hangzhou 310058, China; 22113002@zju.edu.cn (W.Y.);; 2 College of Mechanical Engineering, Xinjiang Agricultural University, Urumqi 830052, China

**Keywords:** soft, end-effector, perceptual, sensor

## Abstract

As consumers demand ever-higher quality standards for agricultural products, the inspection of such goods has become an integral component of the agricultural production process. Unfortunately, traditional testing methods necessitate the deployment of numerous bulky machines and cannot accurately determine the quality of produce prior to harvest. In recent years, with the advancement of soft robot technology, stretchable electronic technology, and material science, integrating flexible plant wearable sensors on soft end-effectors has been considered an attractive solution to these problems. This paper critically reviews soft end-effectors, selecting the appropriate drive mode according to the challenges and application scenarios in agriculture: electrically driven, fluid power, and smart material actuators. In addition, a presentation of various sensors installed on soft end-effectors specifically designed for agricultural applications is provided. These sensors include strain, temperature, humidity, and chemical sensors. Lastly, an in-depth analysis is conducted on the significance of implementing soft end-effectors in agriculture as well as the potential opportunities and challenges that will arise in the future.

## 1. Introduction

The global food supply has been impacted by climate change and an increase in the world population, leading to a need for the agricultural sector to double its production by 2050 to meet growing food demands [1]. This task presents a significant challenge as it must be accomplished despite the issues of rural population decline, scarcity of land, and other natural resources [2]. To overcome these difficulties, traditional agriculture must undergo a fundamental transformation. Robotics and autonomous, unmanned farming are considered viable options for future agriculture to create more efficient, safe, and environmentally friendly farms [3].

In the future of unmanned agriculture, end-effectors, as an integral component, will primarily fulfill the tasks of harvesting, sorting, and detecting certain agricultural products [4]. However, these end-effectors present unique challenges compared to other sectors due to the irregularity of product positioning during harvesting, variations in sizes and shapes, and the fragile nature of agricultural products [5]. Additionally, end-effectors for grasping and manipulation applications in the agri-food domain must be robust and precise. 

The traditional fully-actuated rigid end-effector, which controls the same number of driving sources as the degree of freedom to realize a specified pose, has been widely applied in fruit harvesting robots [4]. This tool has successfully improved work productivity and achieved high accuracy, repeatability, and easy maintenance. However, traditional end-effectors still face several limitations in the face of the previously mentioned challenges [6]. Firstly, it is difficult to adapt to different sizes of fruits and vegetables accurately because even slight changes in the driven section significantly affect the final grasp posture under the fully actuated method. Secondly, sizeable random positioning errors in unstructured agricultural environments can lead to grasping failure. Finally, due to traditional end-effectors’ rigid joints and links, external or internal damage to fruits, such as abrasions, cuts, scratches, wounds, creases, and stem punctures, often occurs.

In recent years, significant progress has been made in developing and applying soft end-effectors, also known as soft grippers, due to advances in soft robotics, stretchable electronics, and materials science [7,8]. A bioinspired method has been proposed for the development of soft end-effectors, which have since found use in a variety of fields, such as hazardous operations, industrial applications [9,10,11,12], medical applications like rehabilitation robots [13] and surgical robots [14,15,16], and other areas where they can safely interact within an unstructured environment and deal with uncertain and dynamic tasks, thereby reducing damage to objects [17,18]. Therefore, introducing soft end-effectors into agriculture is necessary since they integrate underactuation and compliance and allow for better adaptation to targets of varying sizes and shapes. Soft grippers, made using hyperelastic materials, replace rigid joints and can accurately adapt to different objects, causing significantly less damage than traditional end-effectors. In addition, they can adapt to an unstructured environment and easily grip products without decomposing the control actions in the complex tasks of agriculture.

Furthermore, as more and more integrated sensors replace traditional bulky, large instruments, this allows soft grips to gain perceptions such as contact force, temperature, surface roughness, and even chemical information about the target [19,20]. This information can guide the actuator to take the correct action by accessing the object’s position, force, and physiological state. Compared with traditional detection methods, using integrated sensors has many advantages. Generally, they are small in size, low in cost, and practical without professional training. Especially for agriculture, integrated sensors can conduct tests on the physiological indicators of products during the growth process, which is difficult to achieve with large-scale detection equipment. However, traditional end-effectors often utilize rigid silica-based electronic sensors that may cause damage to delicate fruits and vegetables. Therefore, researchers have developed flexible and stretchable sensors that can integrate with soft end-effectors to avoid this issue. Recent literature by Li et al. and Wang H. et al. provides a comprehensive understanding of the developments in flexible and stretchable sensors in soft robots with mechanical sensing abilities [21,22]. Furthermore, other scholars have classified stretchable sensors based on different materials and preparation processes, such as printing properties [23], nanomaterials [24], conductive polymers and their composites [25], and conductive polymer nanocomposites [26]. Zhang et al. have also reviewed the use of soft grippers in agriculture, which can help address problems related to mechanical information detection [27]. However, detecting chemical information is equally crucial in the agricultural field. Therefore, it is essential to summarize the applications of flexible and stretchable sensors that can detect both physical and chemical data.

Of course, the emergence of soft end-effectors is not intended to replace currently mature production technologies, such as mechanized rice planting and harvesting, etc. [18]. Instead, it aims to achieve certain functions that traditional agricultural machinery cannot accomplish, such as non-destructive fruit picking and sorting [4]. Therefore, we need to select suitable soft end-effectors based on their application scenarios and the agricultural challenges they can overcome.

**Figure 1:**
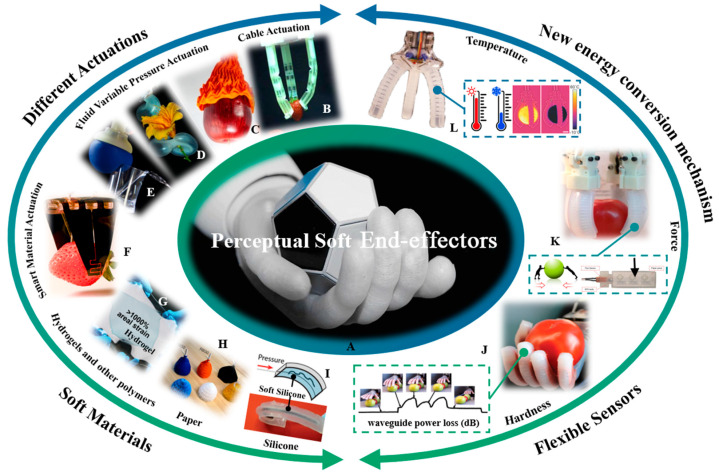
Essential elements of the perceptual soft end-effectors. The combination of soft materials, different actuators, and flexible sensors with a new energy conversion mechanism can achieve a soft end-effector like a human’s hand. (**A**) Agricultural end-effectors in the future. (**B**) Cable-driven gripper [28]. (**C**–**E**) Grippers driven by variable fluid pressure [29,30,31]. (**F**) Grippers driven by smart materials [32]. (**G**–**I**) Soft materials include hydrogels [33] and other polymers, paper [34], and silicone [31]. (**J**–**L**) Soft gripper embedded with a flexible sensor can measure the softness [35], contact force [36], and temperature [37] of fruits and vegetables.

The primary objective of this review is to extract knowledge from existing research on soft end-effectors and sensors, serving as a reference for individuals who may want to employ the driving method and sensors of soft end-effectors in the agricultural field, as illustrated in Figure 1. This article presents a comprehensive overview of soft end-effectors according to the challenges existing in agriculture and introduces the different driving methods: electrically driven actuators, fluid power actuators, and smart material actuators. Moreover, a comprehensive introduction is provided regarding the various sensors installed on soft end-effectors in the field of agriculture, including strain, temperature, humidity, and chemical sensors. An extensive discussion is presented concerning the key opportunities and challenges that soft end-effectors bring to the agricultural field, which researchers and practitioners may encounter in the future.

## 2. Soft End-Effector

Currently, several types of end-effectors are widely used in agriculture [4]. They are designed to overcome some challenges in agricultural production. First, they address the variability of fruits, including their shapes, sizes, maturity indicators, growth characteristics, and stem strength [3]. For example, fruits that grow in clusters like grapes and cherries cannot be effectively harvested through direct gripping, unlike single-grown fruits like apples and pears [38]. Apart from that, cable-driven soft end-effectors are suitable for harvesting small-sized fruits, while fluid pressure-driven ones can be used in a wrap-around manner to harvest larger-sized fruits.

Secondly, end-effectors overcome limitations in picking positions [39]. Fruits can grow both above and below the ground, with above-ground growth including ground-level, vine, and tree growth. The height of the fruits has a significant impact on the design of the driving method and structure of the end-effector [40].

Of course, traditional end-effectors in harvesting processes may cause damage to agricultural products due to rigid contact [5]. Such damage directly affects the quality of agricultural products and increases production costs. However, the use of soft materials as a buffer layer between the end-effector and agricultural products can effectively reduce the probability of damage [41]. Therefore, this chapter classifies soft end-effectors based on the driving method according to agricultural tasks, including electrically driven actuators, fluid power actuators, and smart material actuators. Table 1 presents the soft end-effectors utilized in agricultural production, along with their respective advantages and, disadvantages and the types of agricultural products to which they are applied. However, the smart material end-effector is not included in the table due to its limited carrying capacity and limited application scope.

### 2.1. Electrically Driven Actuator

**Figure 2:**
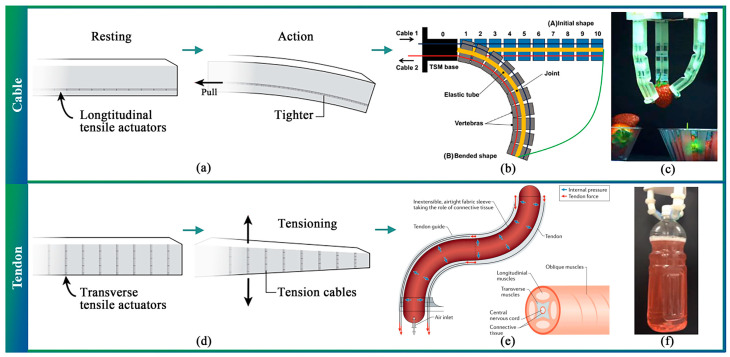
Working principle and application of cable-driven soft end-effector. (**a**,**b**) Principle of cable-driven actuation [58,59]. (**c**) Cable-driven soft end-effector for adaptable and effective grasping [28]. (**d**) Principle of tendon-driven actuation [58]. (**e**,**f**) Tendon-driven soft end-effector [60,61].

During the fruit-picking process, based on the inspiration derived from living creatures and their anatomical parts like elephant trunks [42], Octopus vulgaris [43], and fish [44], cable-driven as a representative of the electric-drive pattern underactuated structures are widely utilized in soft grippers. Electrically-driven actuators primarily adopt a gripping mechanism for harvesting, which mainly targets the peduncle of the fruits. They are typically suitable for harvesting small fruits (Figure 2c). This section presents a comprehensive overview of the soft effectors that employ cable-driven mechanisms with soft materials.

As shown in Figure 2a, the principle of cable actuation, through which the gripper fingers generate a specific bending momentum and force via cable pulling (Figure 2b). In comparison to traditional grippers, cable-driven grippers exhibit improved flexibility and adaptability and can easily cater to the diverse sizes of products with straightforward control. As a result, soft grippers have been successfully employed in various fields. For instance, in the aerospace industry, soft grippers can be used to manipulate and capture target objects. NASA utilized the tendril to extend deep into crevasses for object inspection [45]. Scholars have proposed end-effectors that can reduce the huge impact of spacecraft as they capture space material [46]. Although the working environment is different, it is ultimately composed of flexible materials and deformable structures [47] and provides controllable grasping force and precise operation ability [48], which is a certain inspiration for application in agriculture. In the medical field, robotic cable-driven gloves [11,49,50,51] were created to enhance the movement and coordination of gripping exercises. In addition, cable-driven grippers are suitable for performing minimally invasive surgery [14] by reaching the surgical target due to their entire length actively interacting with the biological structures [52,53,54]. Cable-driven soft grippers are currently used in agriculture, and studies have demonstrated that they are gentle on farm products [28,55,56,57]. 

However, soft torsos have proven challenging to adapt to certain applications, such as gripping small objects. Consequently, scholars have proposed tendons that simulate biological arms’ longitudinal, transverse, and oblique muscles, as depicted in Figure 2d. By activating longitudinal and transverse muscle groups simultaneously, antagonistic muscles can achieve a controlled effect [60,62] (Figure 2e). Ham et al. presented a soft, variable-stiffness gripper that controlled stiffness by pulling tendons. The stiffness of the gripper was noted to be 5.6 times higher than that of a gripper without the counterpart [61] (Figure 2f). 

Even though tendon-based continuum designs can deliver relatively high force, slack and backlash are inevitable. To address this issue, many scholars have initiated antagonistic tendon pairs using some hard materials [63,64] or a disc with a permanent magnet [65] to absorb slack and reduce bending. By optimizing the structure of the soft end-effectors, they can be used to harvest some specific agricultural products, as detailed in Table 1.

### 2.2. Fluid Variable Pressure Actuator

#### 2.2.1. Gas or Liquid as the Driving Medium

The fluid variable pressure actuator is commonly used as an enveloping end-effector that acts on the outer surface of the fruit [66]. After enveloping the fruit, it achieves separation by pulling or twisting off the peduncle [38]. Based on the contact area, it can be classified into three-point style, three-line style, and double-sided style enveloping. This type of actuator is typically used for harvesting larger fruits.

The approach of fluid variable pressure actuators has been proposed by scholars inspired by animals with hydrostatic skeletal structures, such as warts. The principle of the actuator involves applying pressure to different cavities composed of materials with varying elastic moduli to produce deformation and movement in specific directions to grip objects. 

**Figure 3:**
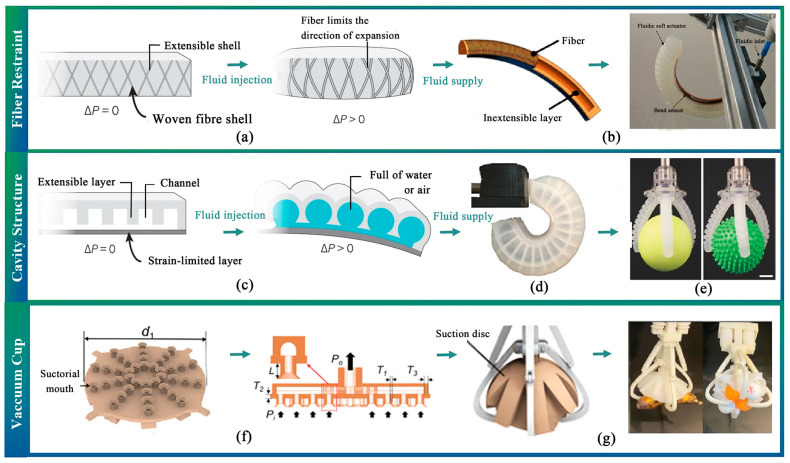
Working principle and application of variable fluid pressure driven soft end-effector (gas or liquid as the driving medium). (**a**) The direction of fiber restraint methods [58]. (**b**) A soft end-effector deforms by twisting a fiber in the cavity [69]. (**c**–**e**) Realizing the deformation of a soft end-effector by designing a closed cavity structure with different parts having different structural stiffnesses [36,37,58]. (**f**,**g**) Improved soft suction disc structure enhances grasping adaptability.

There are two ways to change the pressure in the actuator cavity. One way is to use the fluid to change the internal pressure of the well-designed closed-cavity structure. The fiber restraint limits the expansion of the sealed cavity in a specific direction so that a different degree of distortion can be attained, as shown in Figure 3a. For instance, Deimel et al. created a compliant robotic hand with an excellent payload-to-weight ratio, and it could lift objects almost three times their weight [67]. Furthermore, some scholars have demonstrated that the soft gripper can realize reliable grasping performance without feedback [68,69] (Figure 3b). Similarly, some researchers have designed soft grippers that induce specific bending, twisting, and extending trajectories under pressurization [70,71]. 

The second method involves designing a closed cavity structure with flexible materials to accomplish the deformation of a finger joint [72,73,74,75]. The principle of this method is illustrated in Figure 3c, where a soft actuator whose extensible layer contains several chambers connected by a single channel. The most compliant regions display the lowest stiffness when pressurized [76]. Subsequently, an actuated network of channels can generate complex shapes in elastomeric structures capable of adapting to various object forms [36,37,77] (Figure 3d,e). As a result, some scholars have presented novel soft elastomeric grippers that enable 3D motion [78,79]. The actuator of multiple channels has a synergistic effect on bending, and the coupled pressurization of different media [80,81] can bend the tentacle along any axis and generate 3D motion [29]. However, these methods exhibit relatively slow operation speeds compared to cable grippers because the pressurization and decompression processes entail some time lag. Moreover, the clamping forces that they provide are relatively small. 

Researchers have developed soft vacuum grippers (SVG) to address these issues using the forces created by the local vacuum generated between the target surfaces and the soft grippers. However, conventional vacuum grippers often fail when contacting irregular and rough surfaces due to air leakage. Song et al. solved this problem by utilizing a thinner and softer suction cup that adapts to the target’s rough surfaces and enhances the airtightness of the soft vacuum gripper [82]. Additionally, Wu et al. combined multiple suction cups and a package structure to increase the adaptive grasping of objects [83] (Figure 3f,g). 

Hydraulic and pneumatic-driven soft end-effectors have been widely applied in agriculture, particularly pneumatic-driven ones. This is because their system design is simpler compared to hydraulic-driven end-effectors, and the load capacity can meet the requirements of most agricultural products. Among pneumatic-driven soft end-effectors, the most common approach is to utilize a cavity structure component for harvesting agricultural products [84,85,86,87]. Suction cups are also a simple and practical type of soft end-effector. For instance, Park et al. employed a combination of blades, suction cups, and bagging for harvesting tomatoes [88]. Additionally, considering the significant variation in size and shape of agricultural products, Edurdo et al. designed a modular and deformable soft gripper to handle objects of different sizes and shapes [89]. In the future, pneumatic-driven soft end-effectors will play a vital role in liberating labor in agricultural production.

Moreover, the air supply system is one of the most critical components of the pneumatic actuator. The traditional gas supply method uses compressed gas stored in large tanks, limiting its use in some unstructured agricultural environments requiring autonomous walking, such as terraced fields and hillsides, due to their large volumes and weights. Scholars have proposed several integrated small drive systems that use a battery to power a micropump [90,91,92]. Some scholars have even driven soft robots using chemical reactions such as the instantaneous combustion of methane or butane to generate energy and move the robot [93,94]. An adjustable battery-driven device using the principle of hydrogen peroxide decomposition was developed [95]. However, air supply systems are still under development.

Scholars have introduced using liquids instead of gases to drive the soft gripper, resulting in a relatively faster operation speed. Liquids are incompressible, and the corresponding soft grippers can operate at high frequencies [96,97]. 

In addition, researchers have proposed bionic soft actuators to enhance the performance of grippers. For instance, Hoang et al. proposed a helical soft-fabric robotic gripper with stiffness adjustability and sensory feedback inspired by elephant trunks. It features a unique structure for high load capacity and can grasp objects of varying geometries and weights, up to 220 times its mass [98]. Hoang’s team further proposed a soft robotic fabric gripper that incorporated a bio-inspired gecko adhesive and a thermo-responsive variable stiffness filament. Experimental studies have shown the optimized gripper’s holding power to be approximately five times higher.[99]. In achieving high-speed grasping, researchers have considered emulating the flycatcher’s predation strategy by employing a pressure-responsive approach for rapid predation [100]. For example, inspired by this distinctive stress-response approach, some researchers developed a high-speed soft gripper that could sense mechanical stimuli and actuate instantly. It allows for repeatable and controllable trigger processes [101]. These biomimetic soft actuators exhibit larger clamping forces or faster response times than pre-optimized actuators, but their manufacturing process is generally more complex. 

#### 2.2.2. Granular Material as the Driving Medium

For irregularly shaped agricultural products such as potatoes, cucumbers, etc., which are often multi-piece or elongated, the previously mentioned soft end-effector may not be effective in grasping them [102]. However, an actuator that uses granular material as the medium can effectively solve this problem.

**Figure 4:**
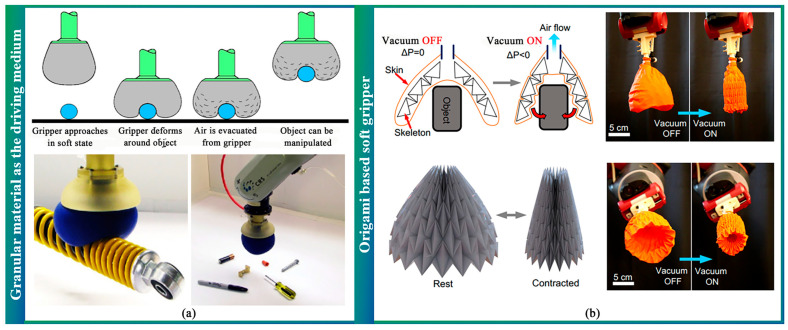
(**a**) Working principle and application of variable fluid pressure-driven soft end-effector (granular material as the driving medium) [30]. (**b**) Working principles of the origami “magic-ball” skeleton and a gripper prototype from different perspectives [104].

Granular material comprises a vast collection of closely packed solid particles surrounded by a gas or liquid that can flow around a target and conform to its shape when pressed onto it. By altering fluid pressure, the granular material can rapidly contract and harden, pinching and holding the object without sensory feedback [30]. Some researchers have designed soft grippers with granular materials to achieve excellent adaptability and flexibility in grasping irregular and fragile targets. In this approach, individual fingers are replaced by a single mass of fine material that flows around and conforms to the target’s shape [30], as shown in Figure 4a. Scholars have introduced sensing systems enabling the gripper to identify grasped objects, improving gripping and manipulation performance [103]. Furthermore, an origami-based soft gripper, similar to a granular jamming gripper, has been developed. For instance, Li et al. proposed a soft gripper that could envelop an entire object or part of it for grasping and manipulation [104], as shown in Figure 4b.

**Figure 5:**
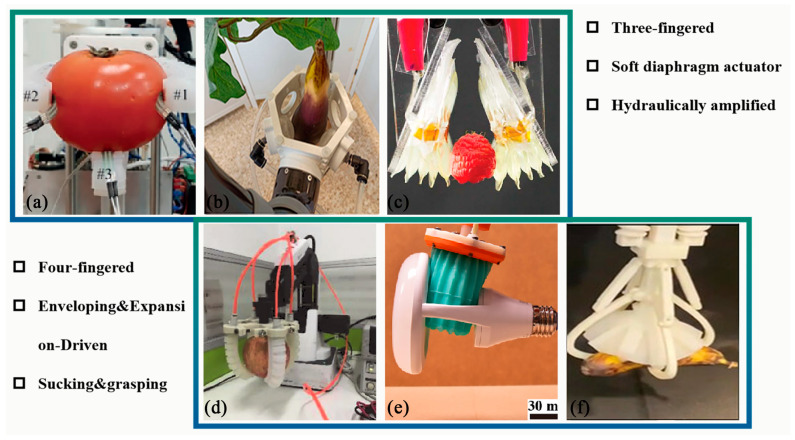
Application of fluid variable pressure actuator. (**a**) A soft robotic three-fingered gripper [121]. (**b**) A soft gripper based on modules that combine a pneumatic-driven soft diaphragm actuator and a 3D-printed structure [89]. (**c**) Hydraulically amplified self-healing electrostatic (HASEL) actuators [122]. (**d**) A soft robotic four-fingered gripper [123]. (**e**) A multimodal, enveloping soft gripper [124]. (**f**) A soft gripper inspired by the glowing sucker octopus [83].

Additionally, the granular jamming driving method can also be applied to the cable-driven gripper to increase its softness [105]. Akashi et al. designed the granular jamming and vacuum-controlled adaptable gripper to achieve the gripping of fruits [102]. However, applying a vacuum to a fluid variable pressure-driven gripper leads to the collapse of the membrane on the filling material, increasing the density and impeding the relative motion of the particles, thus increasing the system’s rigidity. This approach ensures that the finger does not buckle during operation [106]. For example, Al Abeach et al. designed a soft gripper that could increase stiffness by 235% [107]. This method requires a target with a large surface roughness. Some of the fluid pressure actuators used in agriculture are shown in Figure 5.

**Table 1:** Summary of the application of soft end-effectors in agricultural production.

Drive Mode	Structure	Pros	Cons	Applications
**Electric**	Cable	High flexibility and versatility, enabling a wide range of motion control. Lightweight and easy to integrate into various applications.	Susceptible to the elasticity and expansion of cables, which may introduce mechanical errors. Greater friction and wear can affect its lifespan and reliability.	Cron [108], pear, orange, peach, tomato [109], strawberry [110].
	Tendon	High precision and accurate control capabilities. The relative simplicity of the tendons makes them easy to maintain and adjust.	Complex tendon design and layout, require precise adjustment and anchoring. Limited degrees of freedom and motion range.	Watermelon [111], mushroom [112], banana [113], apple [5].
**Hydraulic**	Liquid	High load-bearing capacity and precise motion control capabilities. Good force transmission efficiency and stability. Easy control and stability of liquid mediums.	Requires a liquid supply and control system, resulting in increased complexity. The possibility of leaks and oil spills leads to environmental issues and maintenance challenges. Certain limitations for high-speed motion and quick response applications.	Apple [114], Cherry tomato [84].
**Pneumatic**	Gas	Quick response and high-speed motion capabilities. Ability to achieve significant force and load-bearing capacity. Simple pneumatic system and control regulation.	Requires a source of gas and a pneumatic system, which may be sensitive to environmental conditions. Requires higher maintenance and verification of pneumatic components and pipelines. Lower motion accuracy is influenced by gas pressure and flow fluctuations.	Strawberry [115], tomato [88], Lemon [116], pomegranate, Melon [89], Plum [85], cherry, apple [87,117,118], banana [119].
	Granular material	High adaptability and flexibility, with easily accessible and adjustable materials. Ability to achieve adaptive shapes and deformations.	Requires careful selection and control of granular materials, considering particle size requirements. Relatively lower motion accuracy and force transmission efficiency.	Apple, banana [102].
	Origami	Lightweight and compact design. Customizable shape and size. Mechanical robustness.	Fabrication complexity. Limited actuation range. Sensitivity to external factors.	Tomato [120], apple [104], Strawberry

### 2.3. Smart Material Actuator

The field of smart material actuation has recently emerged as a means of controlling the deformation of advanced materials by applying various field effects (such as electric, thermal, or magnetic fields). Among the materials utilized in this method are shape-memory materials (SMMs), electroactive polymers (EAPs), and stimulus-responsive hydrogels, which are considered state-of-the-art. These smart materials have the potential to significantly impact various technologies, particularly in the fields of automotive, aerospace, and biomedical applications, as well as in the realm of soft agricultural end-effectors [125]. In this section, the implementation of smart materials in the development of soft end-effectors is explored.

In the field of agriculture, the fixation of certain flexible sensors can be achieved using these self-adaptive materials. Additionally, for small-sized and low-mass agricultural products, smart material actuators are more suitable and cost-effective compared to actuators constructed with ordinary materials.

#### 2.3.1. Shape Memory Materials Actuator

SMM grippers, which include both shape memory alloys (SMAs) and shape memory polymers (SMPs), can be manipulated through temperature control and regulated through the adjustment of a current pulse, allowing these devices to be lightweight and manageable without requiring extensive external resources. However, the efficiency of SMA grippers is limited by their relatively slow recovery time from deformation. At the same time, SMPs are unsuitable for operation in harsh conditions due to their susceptibility to environmental factors and overall low efficiency in grasping objects. While SMMs offer greater control over their parameters than other actuation methods, they suffer from lower deformation speeds, limiting their grasping efficiency.

#### 2.3.2. Electroactive Polymers Actuator

**Figure 6:**
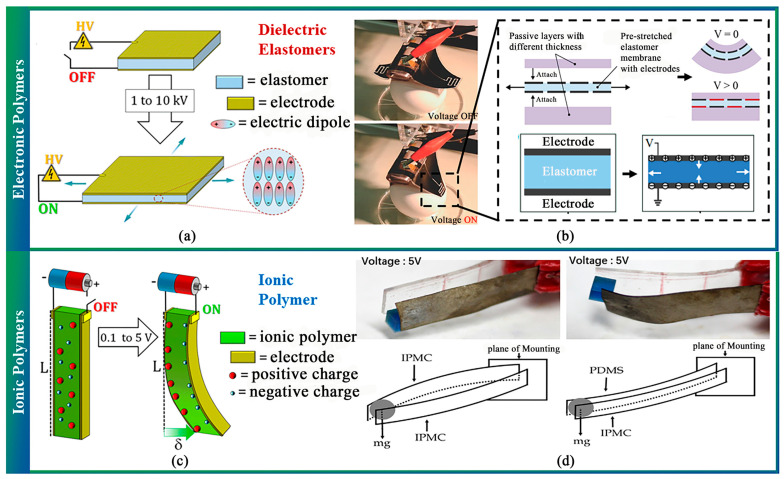
Working principle and application of EAP soft end-effector. (**a**) Principle of electronic polymers [144]. (**b**) DEA gripper [32]. (**c**) Principle of ionic polymers [144]. (**d**) Ionic EAP gripper [142].

Electroactive polymers (EAPs) are smart materials capable of deforming under electric fields, offering advantages such as low density, high actuation strain, softness, and affordability [126]. Dielectric elastomers (DEs), a specific type of EAP, respond to electrostatic charges, enabling high actuation strains, rapid response times, and long lifetimes [127,128]. As shown in Figure 6a,b, dielectric elastomer actuators (DEAs) are achieved by placing a DE film between flexible electrodes, with applied voltage causing thickness reduction and area expansion, allowing strains over 100%. Due to their straightforward working principle, DEs have been integrated into various soft actuators [31,129,130,131,132,133,134,135,136,137]. In agriculture, DEAs are used to stimulate the vibration of pear fruit to evaluate its quality [138]. However, DEAs due to the need for a high driving voltage, there will be an electrical breakdown [126]. Ionic Polymers Actuator, instead, has been proposed as a lower voltage alternative that utilizes polymer gels, ionic polymer-metal composites (IPMCs) [139], conjugated polymers, or carbon nanotubes [140] to achieve mechanical deformations with a driving voltage in the order of 1 V and a reversible process (Figure 6c). However, compared with the former, the response time and lifetime of the IPMC actuator can be limited [141,142,143] (Figure 6d). Through the optimization of materials, EAP actuators are expected to be used in agriculture. 

#### 2.3.3. Liquid Crystal Elastomers

Liquid Crystal Elastomers (LCEs) are novel materials that combine liquid crystal and elastomer properties. Their ordered liquid crystal molecule arrangements and physical characteristics make them ideal for applications like end-effectors and mechanical grippers [145]. LCEs can undergo shape changes through stimuli like temperature and electromagnetic fields, enabling fine-tuning and micro-manipulation. Their flexibility allows them to adapt to different workpiece shapes and sizes. In the field of mechanical grippers, LCEs have been used to achieve complex movements and detailed control [6,7,146]. However, challenges such as preparation, stability, and standardization need to be addressed for wider practical applications. Continued research is crucial to overcome these challenges and promote the use of LCEs.

#### 2.3.4. Stimulus-Responsive hydrogels

Hydrogels are versatile materials that can deform in response to stimuli such as temperature, humidity, pH, light, electric and magnetic fields, and pressure, making them ideal for innovative actuation [147]. For example, by introducing stimuli like temperature or pH transitions, hydrogels can produce powerful forces on surrounding objects [148]. Stimuli-responsive grippers have shown impressive load-to-weight ratios. Differences in swelling behavior between charged chitosan and cellulose layers have enabled the performance of soft grippers [149]. Hydrogel actuators embedded with superparamagnetic iron oxide nanoparticles can undergo flexible and reversible shape deformations controlled by a magnetic field [150]. Hydrogels offer biocompatibility and biodegradability, making them suitable for biomedical applications. However, issues of low rigidity and instability can be addressed through the use of dynamic covalent bond systems [151,152,153]. More details can be found in this review [154].

In summary, despite the various advantages of smart material actuators, structural and material constraints hinder their widespread use in agricultural production. As a result, further development and optimization of these materials are necessary.

## 3. Sensors

Real-time physical, biological, and chemical sensing technology is essential for testing agricultural products accurately. The emergence of miniaturized analytical instruments such as flexible sensors has made fast, non-destructive testing of agricultural products possible by merely installing them on the end-effector. Miniaturized sensors are now essential components of various mechanical systems, including robotics, as they augment the system’s intelligence [155,156]. In telemedicine, soft sensors that can detect the stiffness of organs or tissues are used to provide tactile enhancement and assist medical personnel in determining the position and depth of lesions [157].

In agricultural production, the quality of agricultural products (size, ripeness, pest damage, pesticide residues, etc.) usually needs to be assessed during post-processing [41]. Firstly, post-processing requires a significant amount of mechanized equipment, which may cause secondary damage to agricultural products during the handling process. Secondly, if any issues are found during post-processing, the affected agricultural products can only be discarded, which increases production costs to a certain extent [8]. By incorporating responsive sensing devices in soft end-effectors, real-time quality detection of crops can be performed during harvesting. This enables timely growth regulation based on the detected information and direct sorting based on sorting requirements. Consequently, the number of post-processing steps is reduced, leading to increased efficiency [36]. The final output from sensors is typically displayed in the form of electrical signals. Therefore, various devices such as oscilloscopes, multimeters, spectrum analyzers, and computers are used to collect these electrical signals and interpret the physical and chemical information obtained by the sensors. This section focuses on sensors installed on soft grippers in agriculture, including strain, temperature, humidity, and chemical sensors. Additionally, we discuss their potential applications and limitations.

### 3.1. Flexible Strain Sensors

The detection of agricultural product appearance, which includes properties such as hardness, volume, shape, and quality, presents a challenge to traditional instruments and techniques due to their limited functionality and complexity. However, the implementation of flexible pressure sensors offers a swift solution for agricultural product quality screening. By converting the aforementioned properties into electrical signals, flexible pressure sensors allow for rapid reflection of agricultural product appearance. Typically, flexible strain sensors consist of electrically conductive sensing films coupled with flexible substrates. When sensing structures are stretched or compressed, microstructural changes in the sensing parts lead to corresponding changes in electrical resistance, capacitance, or light loss. Flexible and stretchable strain sensors can be classified into different types, such as conductive material-type, liquid metal, piezoresistive, piezoelectric, triboelectric nanogenerators, and optical sensors, as outlined in Table 2. In this section, representative sensors are introduced to assist in the future selection of suitable strain sensors for soft end-effectors in the agricultural field.

**Figure 7:**
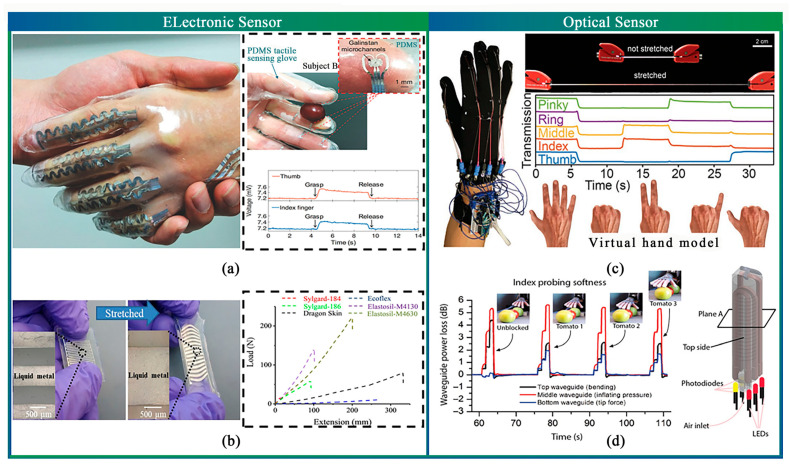
Flexible strain sensors. (**a**) Microfluidic tactile diaphragm pressure sensor based on embedded Galinstan microchannel [167]. (**b**) Stretchable sensors inject liquid metal into microchannels of silicone elastomers [166]. (**c**) Stretchable thermoplastic elastomer optical fiber strain sensor [178]. (**d**) A stretchable optical fiber strain sensor can be embedded in a soft-end effector, allowing the soft hand to perceive [35].

The resistance or capacitance of conductive materials changes under strain, and conductive material sensors are selected based on this principle to measure bending, stretching, and stress once embedded in a silicone body. However, these sensors’ elastic modulus is typically higher than the silicone material’s, leading to restricted movement of the soft end-effector. To address this issue, new materials and processing techniques such as soft lithography and injection molding of conductive Eutectic gallium-indium (EGaIn), a moldable liquid metal, into the microcavity of the silicone body have been explored [159,166,167] (Figure 7a,b). Thin elastomer layers are patterned with microfluidic channels, which are subsequently filled with a liquid conductor such as gallium-containing alloys [168] and conductive carbon grease. Geometric and resistive changes in sensor channels occur due to deformation, enabling strain measurement by measuring the resistive change. Adjusting the channel geometry changes the conductive material sensors to measure different strains, including tensile, shear [160], or curvature [169] strains.

Piezoresistive and piezoelectric materials are common in detecting the strains induced by structural vibrations in macroscale structures. The piezoresistive effect of the piezoresistive materials describes the change in electrical resistance due to external stress or material deformation, which can be frequently measured using Wheatstone bridge circuits [170,171,172]. Piezoelectric materials convert mechanical energy into electrical energy, enabling them to function as both actuators and sensors. Konishi et al. integrated a soft pneumatic actuator with a flexible strain sensor constructed from liquid metal to detect forces as low as 10 mN via changes in electrical resistance [173]. In the process of apple picking, researchers employed sensors to establish a relationship between gripping force, tension, and servo torque. This approach effectively eliminated potential peel damage that may arise during the picking operation [5]. Additionally, Jintao et al. leveraged data gathered from sensors to achieve fruit classification [109].

Triboelectric nanogenerators (TENGs) are another mode of converting external physical stimuli into electrical energy, generating electronic current through friction. Self-powered pressure sensors have been developed using TENGs, making them ideal sensors for soft end-effectors [174,175,176]. For instance, Zhu et al. have devised a two-stage actuator that has been seamlessly incorporated into a self-powered bionic antenna utilizing triboelectric nanogenerators (TENGs) to realize the robot’s active perception [177].

Optical sensors offer a solution to the challenges of flexibility, hysteresis, fabrication complexity, chemical safety, and environmental instability encountered with electrical sensors. Fiber sensors offer a viable solution for detecting changes in internal light intensity, contact force magnitudes, and bend angles that result from the deformation of a soft gripper from external stimuli. The optical loss value can be used to calculate these changes (Figure 7c) [178,179,180,181,182]. Zhao et al. realized the combination of optical sensors and soft end-effectors to detect the shape, structure, and surface roughness of fruits through research. This is not possible with traditional harvesting devices (Figure 7d) [35]. Furthermore, a group of researchers led by Kong Lingjie of Tsinghua University has designed fiber optic sensors using PDMS to detect physical information [183,184,185]. These optoelectronic strain sensors demonstrate low hysteresis and high signal precision while being easy to fabricate and chemically stable.

While current applications of flexible strain sensors are limited to skin sensing due to their low tolerance for strain, they hold promise for future use in agricultural soft actuators, enabling end-effectors to interact with agricultural products and the environment more naturally.

### 3.2. Flexible Temperature Sensor

Temperature is one of the most vital environmental signals for plants, making it necessary to embed a temperature sensor in a soft-end effector. Flexible temperature sensors generally consist of a thermal element connected to flexible substrates. Changes in temperature result in alterations to the sensing component’s resistance, color, or light loss.

**Figure 8:**
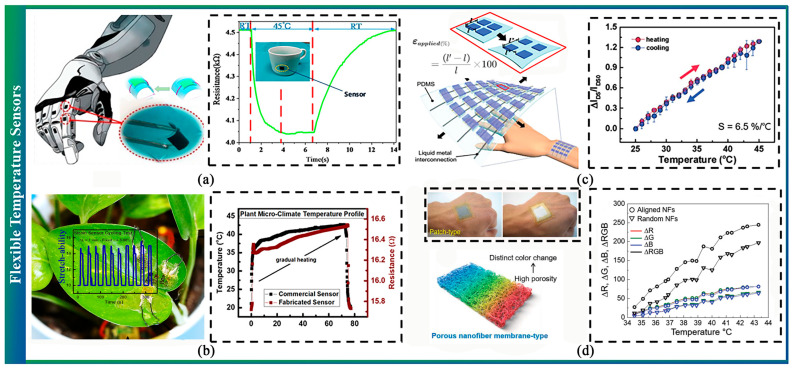
Flexible temperature sensor. (**a**) An adjustable temperature sensor based on reduced graphene oxide [186]. (**b**) Compliant wearable temperature sensor for plant growth monitoring [188]. (**c**) High-sensitivity temperature sensor with thermochromic display [189]. (**d**) Porous Nanofiber Membrane: Rational Platform for Highly Sensitive Thermochromic Sensor [190].

A study by Liu et al. found that r-GO temperature sensors exhibited the most balanced performance compared to single-walled carbon nanotubes and multi-wall carbon nanotubes [186] (Figure 8a). Di et al. similarly discovered that a material made solely from purified plant pectin and crosslinking ions, fabricated in the form of a film, exhibited performance akin to snake pit membranes and outperformed other flexible materials [187]. Wearable temperature sensors developed by Nassar et al. enabled ultralight and flexible sensor designs for plants [188] (Figure 8b). Furthermore, numerous skin-attachable and stretchable high-sensitivity temperature sensors have been demonstrated to date [161,162,163], which can be affixed to soft grippers for testing (Figure 8c,d).

Another method involves using light loss to measure the temperature. Leal-Junior et al. presented the development of temperature sensors based on fiber Bragg gratings embedded in 3D-printed structures made of different materials, namely, polylactic acid and thermoplastic polyurethane [191], and the results indicated that the device demonstrated outstanding temperature sensitivity of 139 pm/°C. Further, the researchers proposed a polymer optical fiber-based temperature sensor [192]. The results indicated that the sensors showed a sensitivity of 1.04 × 10^−3^ °C^−1^ and a linearity of 0.994.

Thermochromic sensors that rely on thermochromic dyes to alter colors based on environmental temperatures have been developed; however, conventional thermochromic sensors exhibit low sensitivity and sluggish response times due to significant dye molecule exposure on the surface [193,194,195]. To address these issues, Kim et al. fabricated a nanofiber membrane that contained thermochromic dyes as a high-sensitivity temperature sensor, utilizing electrospinning techniques. Unlike traditional thermochromic sensors, this sensor improved sensitivity levels by two to fivefold [190]. For a comprehensive evaluation of flexible temperature sensors, please refer to this review [196].

### 3.3. Flexible Humidity Sensor

Extreme humidity can impact crop productivity and plant survival rates, making it important to detect humidity levels for agricultural production. Two types of humidity sensors exist: capacitive and resistive. These humidity sensors are used to detect water produced by plant transpiration, the specific parameters of which are shown in Table 3.

**Figure 9:**
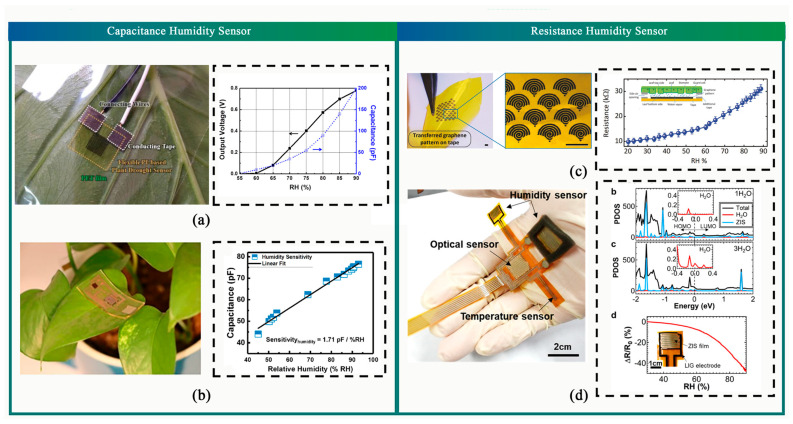
Flexible Gas and PH Sensor. (**a**) Flexible PI-based humidity sensor [197]. (**b**) Flexible, lightweight CMOSenabled multisensory platform [201]. (**c**) Graphene-based nanomaterial humidity sensors [199]. (**d**) Multimodal plant healthcare flexible sensor system [200].

Capacitive sensors rely on changes in the dielectric constant caused by variations in plant moisture content. A method for constructing capacitive sensors by forming Ti/Au electrodes on polyimide (PI) films (Figure 9a) has enabled the detection of moisture content changes ranging from 55% to 90% [197]. Additionally, Lan et al. proposed a wearable humidity sensor (Figure 9b) that features laser-induced graphene technology. The sensor exhibits high sensitivity (3215.25 pF/% RH) and long-term stability (<1% change) [198]. Capacitive humidity sensors provide high sensitivity and a broad detection range; however, they cannot easily detect large objects due to their dual-pole plate structure.

Resistive humidity sensors circumvent the issues encountered by capacitive sensors by leveraging the inherent absorption properties of materials. When these materials come into contact with water, resistance changes occur, resulting in a reflection of surface humidity levels. Oren et al. successfully constructed a humidity sensor (Figure 9c) that detected water transport from maize roots and leaves [199]. An integrated multimodal flexible sensor system for plant growth management was proposed by Lu et al. using stacked ZnIn2S4 (ZIS) nanosheets as a core sensing medium, which exhibited consistent performance in monitoring humidity levels (Figure 9d) [200]. While resistive humidity sensors are less sensitive than capacitive sensors as they absorb water directly, object thickness does not affect their performance.

Furthermore, multimodal flexible sensors with the ability to detect humidity have been developed [201,202]. However, their functions are primarily based on the two methods outlined above. These sensors are projected to be utilized alongside soft actuators to detect humidity levels on plant surfaces throughout production operations.

**Table 3:** Summary of Humidity Sensors.

Sensing Material	Sensing Mechanism	Fabrication Methods	Sensor Performances
Ti/Au [197]	Capacitance	Spin-coating, screen printing, mental sputtering, Reactive ion etching (RIE)	low power-power consumption
Pi [201]	Capacitance	photolithography, spin coating, oxygen plasma, metal sputtering, etch	1.71 pF/%RH
GO [198]	Capacitance	Laser-induced scribing drop-casting	High sensitivity (3215.25 pF/%RH), long-term stability (variation < 1%)
Graphene [199]	Resistance	Soft lithography	0.29 kΩ/%RH
SU-8 photoresist SiO_2_/Si [188]	Resistance	photolithography techniques	repeatedly and reversibly for over one week
PI [202]	Resistance	Metal sputtering	95% reduction
ZnIn2S4 (ZIS) [200]	Resistance	Laser-induced scribing, drop-casting, screen printing, Inkjet-printing	0.0056/%RH

### 3.4. Flexible Chemical Sensor

**Figure 10:**
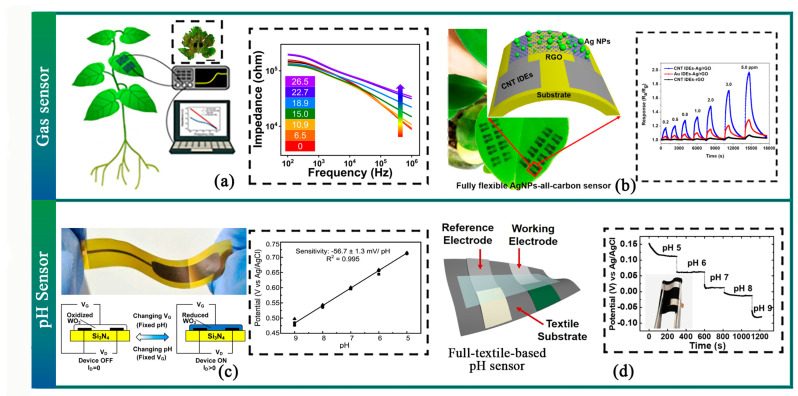
Flexible Gas and PH Sensor. (**a**) A vapor-deposited conducting polymer tattoo can detect ozone damage in fruiting plants [204]. (**b**) A wearable chemical sensor for detecting NO_2_ uses fully flexible AgNP-all-carbon nanostructures [206]. (**c**) WO_3_ nanoparticle-based conformable pH sensor [207]. (**d**) A fully textile-based skin pH sensor [210].

A chemical sensor is an autonomous analytical device that can provide information on the chemical composition of its environment, including gases, ions, electrolytes, and concentrations. Within agriculture, the assessment of gases, pH levels, pesticide residues, and volatile organic compounds (VOCs) is critical since these indicators reflect the quality of produce. Currently, chemical sensors transform various substances into electrical signals, either quantitatively or qualitatively. In gas detection, Hartman et al. developed an optic sensor capable of monitoring levels of NH_3_ [203], which functions by tracking refractive index changes arising from a reversible chemical reaction on the waveguide surface. Kim et al. designed a polymer tattoo capable of detecting O_3_ using a vapor-deposited conductive polymer. The device monitors a refractive index change resulting from a reversible chemical reaction on the waveguide surface. In addition, Kim et al. have designed a polymer tattoo capable of detecting O_3_ using a vapor deposit of conductive polymers [204] (Figure 10a). Additionally, scholars have presented flexible chemical sensors that detect NO_2_ [205,206,207] (Figure 10b). Nakata et al. developed a WO_3_ nanoparticle-based conformable pH sensor [208]. Furthermore, Nakata et al. developed a high-sensitivity wearable pH sensor based on a flexible charge-coupled device [209].

**Figure 11:**
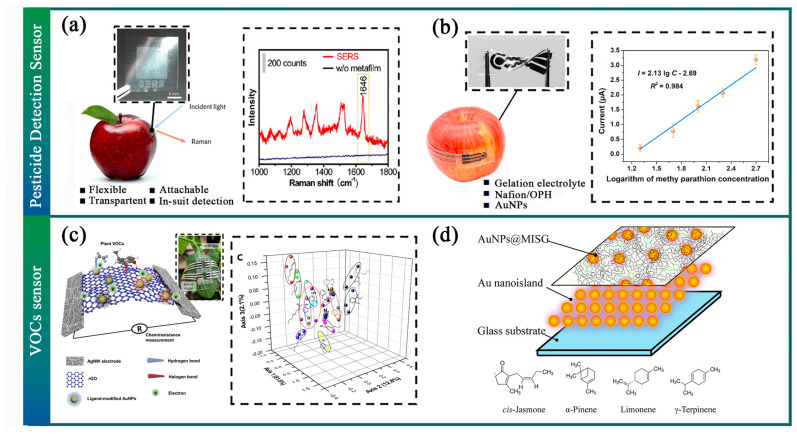
Pesticide Detection and VOCs Sensor. (**a**) A flexible and transparent surface-enhanced Raman scattering (SERS)-active metafilm [210]. (**b**) A biosensor for in-situ pesticide analysis [211]. (**c**) A wearable sensor can detect plant stresses via in real-time chemiresistive profiling of leaf volatiles [215]. (**d**) A localized surface plasmon resonance sensor array to recognize plant biomarkers [216].

Moreover, a flexible chemical sensor has been developed to detect pesticide residues directly on agricultural product surfaces based on the surface-enhanced Raman scattering (SERS) effect. A flexible and transparent SERS-active metafilm capable of revealing chemical residues on surfaces has been proposed [210]. The SERS metafilm can be conformably attached to agricultural product surfaces to amplify analyte Raman signals (Figure 11a). Electrochemical detection is also a viable method for pesticide detection; Zhao et al. developed a wearable biosensor for in-situ pesticide analysis that successfully detects methyl parathion on crop surfaces (Figure 11b) [211]. Moreover, the Electrochemical Impedance Spectroscopy (EIS) test in electrochemical detection can be used to assess the ripeness of agricultural products. Combining the pneumatic gripper with bioimpedance measurement sensors enables non-destructive food quality inspection [86]. 

The detection of volatile organic compounds (VOCs) in plants is a promising method of reflecting their living conditions. When faced with environmental stress, plants often emit VOC signals. Some researchers have already begun using VOCs to monitor pests, diseases, and physical damage within the agricultural industry [212,213,214] (Figure 11c,d). 

To summarize the sensing methods discussed above, while soft sensing technology has made some notable advances, it remains at an early stage of development. The incorporation of sensors allows soft end-effectors to obtain greater physical and chemical information, enhancing robot control and enabling the acquisition of tactile and physiological data from the surrounding environment.

## 4. Summary and Outlook

The end-effector is a critical component that directly interacts with fruits and vegetables in agricultural robots. As the target objects are specific, the end-effectors must boast appropriate active flexibility, adaptability, and security [18]. Perception is equally vital in the farm field, as it helps researchers better analyze the information of fruits and vegetables, such as the contact force, surface roughness, temperature, humidity, and even chemical information [20]. Therefore, agricultural applications require more adaptable and perceptual end-effectors, prompting researchers to introduce soft end-effectors into farming.

Apart from that, perceptual soft end-effectors are expected to address the urgent issues in current agricultural production:

1. Labor costs: Currently, over 90% of agricultural production requires human involvement, and labor costs typically account for more than 50% of the total cost [217]. Moreover, the aging agricultural workforce is gradually decreasing, leading to further increases in labor costs. The inevitable trend for the future is to replace manual labor with mechanization, and the role of soft end-effectors is to replace human hands in harvesting agricultural products [38].
2. Crop damage: Certain agricultural products such as apples, tomatoes, and citrus fruits are prone to damage during harvesting, compromising their quality and ultimately increasing production costs [121]. During harvesting, it is crucial to protect these agricultural products from collisions and compression. Flexible materials, acting as a buffer layer between the end effector and the agricultural products, can effectively reduce the probability of damage.
3. Real-time agricultural quality detection: The quality of agricultural products (size, ripeness, pest damage, pesticide residues, etc.) usually needs to be assessed during post-processing. However, post-processing requires a significant amount of mechanized equipment, which may cause secondary damage to agricultural products. Additionally, any problematic agricultural products discovered during post-processing can only be discarded, leading to increased production costs. By incorporating responsive sensing devices into soft end-effectors, real-time quality detection of crops can be achieved. This allows for timely growth regulation based on the detected information and direct sorting based on sorting requirements, reducing the number of post-processing steps and increasing efficiency to some extent.

Soft end-effectors need to be designed based on different agricultural tasks due to variations in agricultural environments, such as fruit differences and harvesting locations. The different environments determine the differences in driving methods and structures. Common driving methods include electrically driven, fluid-powered, and smart material actuators, each suitable for different agricultural tasks. Selecting appropriate soft end-effectors ensures work efficiency, simplifies the structure, and reduces costs.

Perceptual soft end-effectors offer a range of improvements. Firstly, they can adapt to targets of varying sizes and shapes without extensive parameter adjustments, leading to minimal damage compared to traditional end-effectors. Secondly, stretchable sensors embedded in soft grippers enable greater autonomy and intelligent use of soft grippers by providing a way for gripper fingers to sense contact or proximity to an object and acquire multiple forms of information about the object, thereby enhancing how the grippers interact with manipulated things. Thirdly, soft end-effectors have a simple structure and a low cost. However, applying soft end-effectors in the agricultural field still presents challenges.

Firstly, in agricultural harvesting equipment, soft end-effectors need to be designed according to the actual size of the target, as they cannot meet all sizes of produce and cannot replace all traditional harvesting methods in unmanned agriculture in the future, such as the harvest of rice, corn, and other crops. The efficiency of soft end-effectors will never be as good as the most advanced combined harvesting equipment. Secondly, among various end-effectors, electrically driven and fluid variable pressure-driven ones require additional bulky auxiliary devices, resulting in lower efficiency compared to traditional end-effectors. Also, smart material-driven end-effectors require specific environmental conditions due to their material properties, making their application in the agricultural domain difficult. Thirdly, although flexible sensors are mature, they are rarely combined with soft end-effectors. In this context, the application of perceptual soft end-effectors in agriculture should focus on reduced damage to produce during gripping, sorted produce based on geometric parameters, and in situ product quality analysis.

In the current agricultural scenario, despite the high efficiency achieved by various machinery, such as plowing, transplanting, and harvesting machines, these processes remain semi-automated, necessitating human intervention. Moreover, certain intricate tasks like picking, sorting, and field management unavoidably require human involvement. Consequently, relying solely on existing agricultural equipment falls short of realizing complete unmanned operations. To accomplish unmanned agriculture, the integration of robots equipped with soft end-effectors emerges as an ideal solution. Soft end-effectors facilitate various functions that conventional machinery cannot achieve, such as operating effectively in complex farm environments and executing precise actions based on plant morphology, thereby reducing waste and minimizing damage. Although current flexible robots may not yet match the performance of traditional agricultural machines in certain aspects, future advancements are likely to introduce fully automated robots equipped with soft end-effectors, thereby liberating humans from centuries of traditional farming labor.

This article provides a critical overview of the broad field of perceptual soft end-effectors, categorizing soft end-effectors based on actuation, either electrically driven, fluid power actuator, or smart material actuator. Furthermore, a description is provided of various miniaturized analytical instrument-type sensors for physical, chemical, and biological crop monitoring that have the potential to be integrated into soft end-effectors in agriculture. In conclusion, this paper discussed the significance of applying perceptual soft end-effectors like human hands in agriculture. With the continuous advancement of technology, perceptual soft end-effectors have numerous applications in daily life, including medical, military, and agricultural areas.

## Figures and Tables

**Table 2:** Summary of strain sensors.

Type of Stain Sensor	Materials	Mechanical Robustness (Stretchability (%))	Gauge Factor	Agricultural Applications
Capacitive sensor [158]	Au/Si/Al/PDMS/PET	/	1.042~0.67 kPa^−1^	Assessing ripeness levels of fruits
Conductive material sensor [159,160]	Eutectic gallium indium or EGaIn	350%	28.6–37.0 mV/N	Grasping force detection
Magnetic sensor [161]	NdFeB/PDMS	/	0.07%/mT	Assessing ripeness levels of fruits
Piezoresistive-type sensor [162]	PDMS/PI/Ag/Au	/	Sponse range (0–54 kPa),	positioning of agricultural products
Piezoelectricity-type sensor [163]	AgNWs/PDMS/ZnO	/	17.82 MPa^−1^ (0–16 kPa) 5.75 MPa^−1^ (16–100 kPa)	Fruit hardness monitoring
Triboelectric nanogenerator [164]	AgNWs/TPU	800%	6 mW/m^2^	Identification and positioning of agricultural products
wearable strain sensor [165]	CNT/Graphite	150%	GF:48 (50%strain) GF:353(150% strain)	Monitor plant growth
Optical fiber-type sensor [35]	ELASTOSIL M 4601	850%	3.23 dB/N	Detecting shape and texture, probing softness, and agricultural product recognition.

CNT: carbon nanotube; AgNWs: silver nanowires; TPU: thermoplastic polyurethane; PDMS: polydimethylsiloxane; PET: polyethylene terephthalate.

## Data Availability

Data sharing is not applicable to this article as no new data were created or analyzed in this study.

## Nomenclature

PneuNets	Pneumatic networks
SMA	Smart material actuation
SMMs	Shape memory materials
SMAs	Shape memory alloys
SMPs	Shape memory polymers
LCE	Liquid crystalline elastomer
UV	Ultra-violet
pH	Potential of hydrogen
3D	Three-dimension
EAPs	Electroactive polymers
DEAs	Dielectric elastomer actuators
IPMCs	Ionic polymer-metal composites
2D	Two-dimension
2.5D	2.5-dimension
DOF	Degree of freedom
PDMS	Polydimethylsiloxane
SERS	Surface-enhanced Raman scattering
D-H	Denavit-Hartenberg
PCC	Piecewise constant-curvature
PZT	Lead zirconate titanate
SBS	Styrene-butadiene-styrene
CNT	Carbon nanotube
ITO	Indium tin oxide
PET	Polyethylene terephthalate.
TENGs	Triboelectric Nanogenerators
